# Automatic Recognition of Seismic Intensity Based on RS and GIS: A Case Study in Wenchuan Ms8.0 Earthquake of China

**DOI:** 10.1155/2014/878149

**Published:** 2014-02-03

**Authors:** Qiuwen Zhang, Yan Zhang, Xiaohong Yang, Bin Su

**Affiliations:** Center for Digital Engineering and Simulation, Huazhong University of Science and Technology, 1037 Luoyu Road, Hongshan District, Wuhan 430074, China

## Abstract

In recent years, earthquakes have frequently occurred all over the world, which caused huge casualties and economic losses. It is very necessary and urgent to obtain the seismic intensity map timely so as to master the distribution of the disaster and provide supports for quick earthquake relief. Compared with traditional methods of drawing seismic intensity map, which require many investigations in the field of earthquake area or are too dependent on the empirical formulas, spatial information technologies such as Remote Sensing (RS) and Geographical Information System (GIS) can provide fast and economical way to automatically recognize the seismic intensity. With the integrated application of RS and GIS, this paper proposes a RS/GIS-based approach for automatic recognition of seismic intensity, in which RS is used to retrieve and extract the information on damages caused by earthquake, and GIS is applied to manage and display the data of seismic intensity. The case study in Wenchuan Ms8.0 earthquake in China shows that the information on seismic intensity can be automatically extracted from remotely sensed images as quickly as possible after earthquake occurrence, and the Digital Intensity Model (DIM) can be used to visually query and display the distribution of seismic intensity.

## 1. Introduction

The attenuation map of seismic intensity is continuous and planar in a very large area. It reflects the strength and change rules of damage intensity caused by an earthquake. It is very useful in the analyses of the extent of damage and the distribution of earthquake disaster [1]. The seismic intensity map is the foundation and key task for seismic disaster assessment, relief, and mitigation. The traditional way of drawing seismic intensity map is mainly by field investigation or based on empirical formulas. However, on the one hand, field surveys need to spend a lot of manpower, material resources and are easy to be affected by traffic and weather. On the other hand, the formula way is too relied on the experiences. So, it is difficult to draw seismic intensity map with high precisions that is suitable to the geological conditions of earthquake disaster region.

The rapid development of Remote Sensing (RS) and image processing technology makes it possible to extract the damage information and assess the disaster losses caused by earthquake [2–4], while the GIS-(Geographical Information System-) based data management and graphics drawing technology provide technical supports to the seismic intensity map drawing and rendering. Based on the integrated application of RS and GIS technology, an automatic recognition approach for seismic intensity has been proposed in this paper. It can automatically recognize and extract the potential damage information from the remotely sensed images of the earthquake disaster area and then quickly generate seismic intensity map without going outside. It can provide effective, exact and quick supports for the assessment, relief, and mitigation of earthquake disaster.

## 2. Principle and Methodology

### 2.1. Framework of the Theory and Technology

The automatic recognition of seismic intensity mainly includes two parts: the extracting of information on seismic damage and the management of the corresponding data. Based on this theory, this paper integrates RS and GIS to recognize seismic intensity automatically, where RS is applied to extract the earthquake damage information, and GIS is used to manage and display the data.

### 2.2. The Definition of Seismic Intensity

Seismic intensity is the degree of strength of earthquake damage. It is closely related to the earthquake magnitude, location, and distance from the epicenter, as well as the geotechnical properties [5]. Intensity and magnitude are two fundamental standards to measure an earthquake. They are two closely connected but different concepts. Magnitude represents the severity of an earthquake, and it is a description of the seismic energy [6]. Intensity indicates the damage degree of an earthquake at various points in a large area. An earthquake has only one magnitude but can have many different intensity values. Intensity is very important for earthquake disaster assessment and earthquake relief.

Seismic intensity is mainly based on the building damage, surface destruction, feelings of people, and so on. Earthquake researchers around the world have developed many different seismic intensity scales. For an example, based on the degree of damages caused by earthquake, the seismic intensity in China is divided into 12 degrees [7].

A lot of points with the same intensity value in a large area can constitute an isoseismal map [8]. In the earthquake disaster assessment, researchers often convert the discrete isoseismal into the continuous seismic intensity attenuation map. Seismic intensity distribution map can help people to master the distribution of earthquake damage and calculate and assess the losses resulted from the earthquake.

### 2.3. RS-Based Automatic Recognition of Earthquake Damage

Remotely sensed imagery can reflect the degree of earthquake disaster. It can be used to recognize the collapse rate of buildings and the changes of the earth's surface through comparing the remotely sensed images before and after the earthquake. Different degree of destructions and losses represents different seismic intensity region. Therefore, through image processing and information extraction, seismic intensity can be identified automatically from the remotely sensed images.

#### 2.3.1. Preprocessing of Remotely Sensed Images

In acquisition process, remotely sensed images are easily affected by time, spectrum, and so forth. This will lead to errors between the images and the complex surface information, which would influence the image interpretation and the accuracy of the extracted damage. Therefore, it is very necessary to preprocess the original images before their utilization. Preprocessing of remotely sensed images mainly includes image correction, registration, and enhancement. After processes of deformation, distortion, blur, and noise, image correction can obtain an image as real as possible in geometry and radiation [9, 10]. After image correction and registration, the coordinates and positions of damage information are more accurate. It makes the collapsed buildings in the image clearer and easier to be recognized. Image enhancement is mainly used to eliminate the noise of the images, improve image visual effect, and highlight the image with earthquake damage information [11, 12]. So it is more easily to recognize the earthquake damages caused by the destruction of buildings and lifeline engineering.

#### 2.3.2. Extraction of Earthquake Damage Information

According to the classification standard of seismic intensity, the information of earthquake damage is generally characterized by buildings and surface destructions. So the RS image-based information extraction of buildings and surface destructions is the premise of seismic intensity recognition. Scholars around the world have made a lot of related researches [13–16]. Currently, the main extraction methods are change detection and information classification. Visual interpretation, supervised classification, unsupervised classification, and object-oriented classification are all the prevailing classification ways. Both the methods of supervised classification and unsupervised classification extract information according to spectral feature in pixels unit. They rarely consider the texture structure and the correlation between adjacent pixels [17–19], so, after the classification, small patches will still exist [20].

The object-oriented classification is a new classification method, which is suitable for remotely sensed images with high resolution. The basic principle is to form the pixels that have the same characteristics of an object according to the pixel shape, color, texture, and other parameters, and then to do classification according to the features of each object. Firstly, the image is divided into several pixel sets, and every pixel set has similar information. Secondly, regarding the pixel set as a basic classification unit, fuzzy classification algorithm is used to generate the probability that the object belongs to a certain class. Finally, the classification results are determined based on the maximum probability.

This paper adopts the method of object-oriented classification to realize the automatic identification of damages from the remotely sensed images. Then, supplement by manual inspection is used to further complete the information recognition and extraction. Firstly, we do the multiscale segmentation of remotely sensed images and then classify each object through the color, shape, texture, and other features. At last, we extract the damage information according to the feature information. After quantitative analyses of the extraction results, we can give the collapse rate of buildings and the earth's surface destruction status, which is very important for the next recognition of seismic intensity.

#### 2.3.3. Recognition of Seismic Intensity

The recognition of seismic intensity is mainly based on the collapse rate of buildings and the earth's surface damage status. After comparing the damage situations with the intensity standard, we can generate the intensity in a region. According to the earthquake damage matrix of Sichuan, Gansu, and Shanxi Province in China, Table 1 gives the complete collapse rate of buildings with three different types of structures. In the experiment, when the intensity value is below six, most buildings are only general or slight damaged, and it is very difficult to identify these damaged houses. So intensity values in Table 1 are all above six.

### 2.4. GIS-Based Seismic Intensity Drawing and Rendering

Each seismic intensity value obtained through remote sensing interpretation only presents the disaster degree in a partial small area. It is a point-shaped and discrete intensity. However, in order to know the distribution of disaster caused by an earthquake in a larger scale, we need to obtain all intensity values in the disaster region. Seismic intensity map is a planar-shaped and continuous intensity. Therefore, it is quite necessary to convert the point-shaped and discrete intensity to planar-shaped and continuous ones.

#### 2.4.1. Digital Intensity Model

In this paper, we introduce the idea of Digital Terrain Model (DTM) in GIS to build Digital Intensity Model (DIM). In DIM, through interpolation of the discrete intensity value, we can obtain the intensity value at any point on the map.

In the view of mathematics, DIM is a sequence of two dimensional function as follows:

(1) $$\begin{array}{c} I_{p}=f_{p}\left(x_{p},y_{p}\right),\;\left(p=1,2,3,\ldots ,n\right), \end{array}$$

where *I*_*p*_ is the intensity value at point *p*; *X*_*p*_ and *Y*_*p*_ are the coordinates of point *p*; *n* is the number of ground points.

#### 2.4.2. Map Drawing and Rendering

In the digital intensity model, every point in the earthquake-stricken area has a seismic intensity value. The continuous and abundant information is convenient for people to quickly assess the earthquake damage and spatial distribution of the disaster. However, the intensity values obtained from the interpretation of remotely sensed images are always discrete and limited. Based on DIM, a mathematical interpolation method has been proposed to draw isoseismal line by using these discrete intensity values. After comparison of several mathematical methods, this study adopts the minimum curvature interpolation method. The interpolation surface generated by the minimum curvature method is similar to rectangular elastic thin films. It covers each point and has a minimum amount of bending. This method not only strictly respects the data but also generates smooth curved surface that is extremely close to the theoretical intensity curve.

Although isoseismal line has realized the conversion from discrete intensity values to continuous ones, however, it is still discrete between the lines. In order to solve this problem, we construct a rendering seismic intensity map, in which different color represents different intensity. It is really a continuous way to express the distribution of seismic intensity.

## 3. Case Study

### 3.1. Studied Area

**Figure 1:**
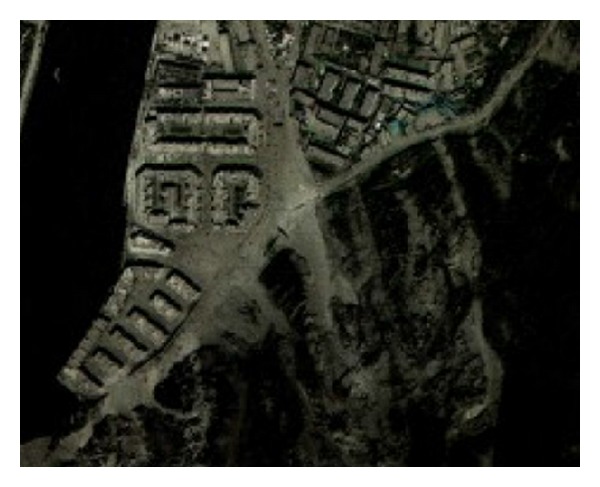
IKONOS remotely sensed image of Wenchuan County.

On May 12, 2008, the Ms8.0 earthquake occurred in Wenchuan County of Sichuan Province in western China. The epicenter intensity was eleven. The epicenter is mainly located at 103.36 degrees of east longitude and 30.98 degrees of north latitude. The focal depth of this earthquake was very shallow and the secondary disasters were serious. In this earthquake, almost seventy thousand people died, and huge economic losses were caused in the disaster areas. It is one of the most destructive and biggest earthquakes in China, even in the world. The remotely sensed IKONOS image of Wenchuan County is shown in Figure 1.

### 3.2. RS-Based Automatic Recognition of Seismic Intensity

Before drawing a seismic intensity map, at first, it needs to obtain a lot of intensity values in the region. Take Wenchuan County as an example, this section gives the method of automatic recognition of seismic intensity.

#### 3.2.1. Preprocessing of RS Images

**Figure 2:**
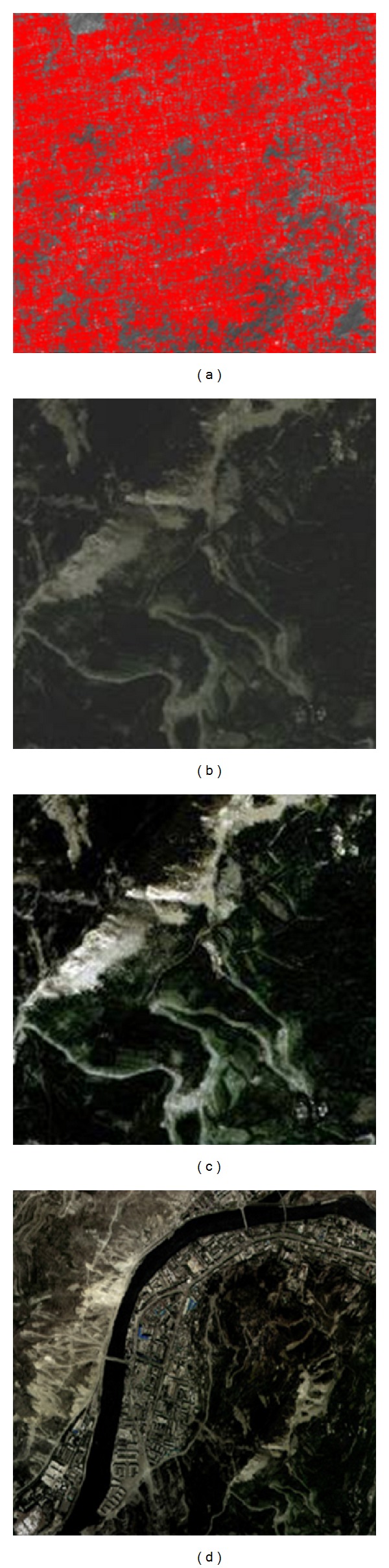
(a) Result image of automatic control point matching; (b) original image before enhancement; (c) Image after enhancement; (d) image after preprocessing.

This study chooses IKONOS satellite remotely sensed image to extract earthquake disaster information. Firstly, using the algorithm of feature matching, we matched the 1 : 10000 orthophotomap and DEM data. The accuracy is consistent with the reference data. This will not only meets the same level of imagery production requirements but also greatly improves the efficiency of access control points.  Figure 2(a) shows the result image of automatic control point matching. The enhancement of remotely sensed image is to highlight the target information so as to make it clearer. Figures 2(b) and 2(c) are images before and after the enhancement. It shows that after enhancement, the image has higher resolution and clearer level, especially for the buildings. It is easier and facilitated for the next interpretation and recognition. After these preprocessing, the remotely sensed image of Wenchuan County could be seen in Figure 2(d).

#### 3.2.2. Automatic Extraction of Damage Information from RS Images

**Figure 3:**
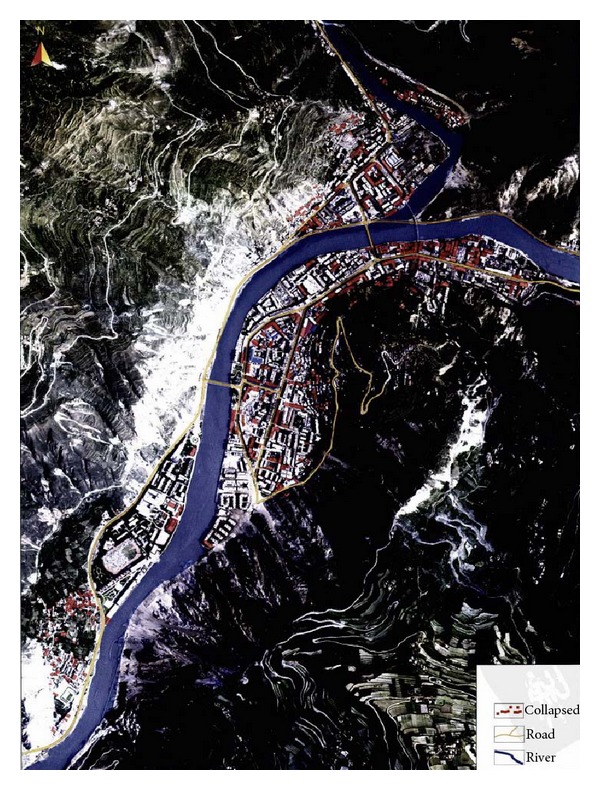
The extraction result of collapsed buildings from RS image.

The object-oriented way to extract the information of destroyed buildings mainly includes two parts. One is the image segmentation used to form image object. The other is the recognition of buildings through the feature of image object. This study adopts four categories to get the final classification result of damage information. Firstly, we extract the nonvegetation information based on Normalized Difference Vegetation Index (NDVI). Secondly, we extract the basic intact buildings in the nonvegetation category. Thirdly, we extract the destructive buildings by the feature of length-width ratio. Lastly, we extract the collapse buildings through the nearest neighbor distance method. After these four steps, there are still a small amount of misclassified cases. It needs to do some manual inspection as a supplement.  Figure 3 shows the extraction result of collapsed buildings from the remotely sensed images in the studied area.

#### 3.2.3. Recognition of Seismic Intensity from RS Images

Based on the preprocessing and damage information extraction above, we can obtain the layer of building damage classification and the layer of building structure classification. After overlaying these two layers, we can get the basic intact rate, destroy rate, and collapse rate of these three types of buildings with different corresponding structures, as well as the destructive situation of the earth surface (Table 2).

After comparison between Tables 1 and 2, it can be concluded that the building damage rate in Wenchuan County is very similar to the tenth degree of seismic intensity area. So the seismic intensity value of Wenchuan County is recognized to be ten.

### 3.3. Seismic Intensity Map Drawing and Rendering Based on GIS

#### 3.3.1. Isoseismal Drawing Based on DIM

Isoseismal line drawing is a conversion from the single and discrete intensity values into continuous intensity line. It needs to obtain several intensity values. In this study, we first choose 15 representative areas to evaluate their seismic intensity values, and the results are shown in Table 3.

**Figure 4:**
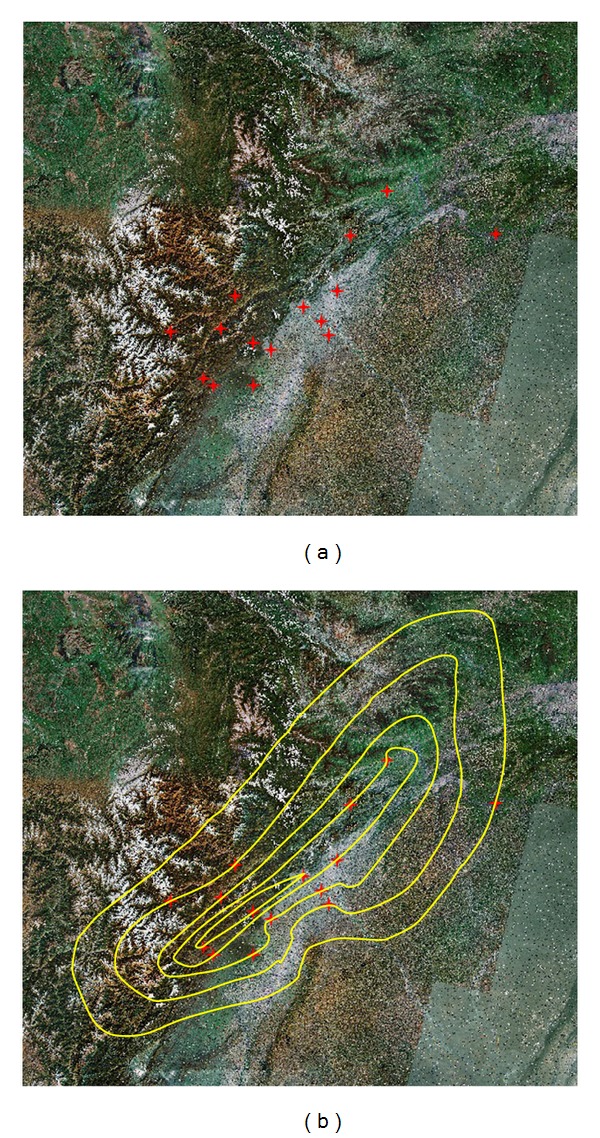
(a) Fifteen points labeled in remotely sensed satellite image; (b) isoseismal line based on the fifteen discrete evaluation areas.

In DIM, each point has three attributions as (*x*, *y*, *I*), in which *x* and *y* are the coordinates like longitude and latitude, *I* is the seismic intensity. Any point with corresponding *x* and *y* can be labeled to the remotely sensed satellite image of the studied area, and then the isoseismal line can be drawn according to the minimum curvature method.  Figure 4 shows the isoseismal line based on the fifteen evaluation areas in Table 3.

**Figure 5:**
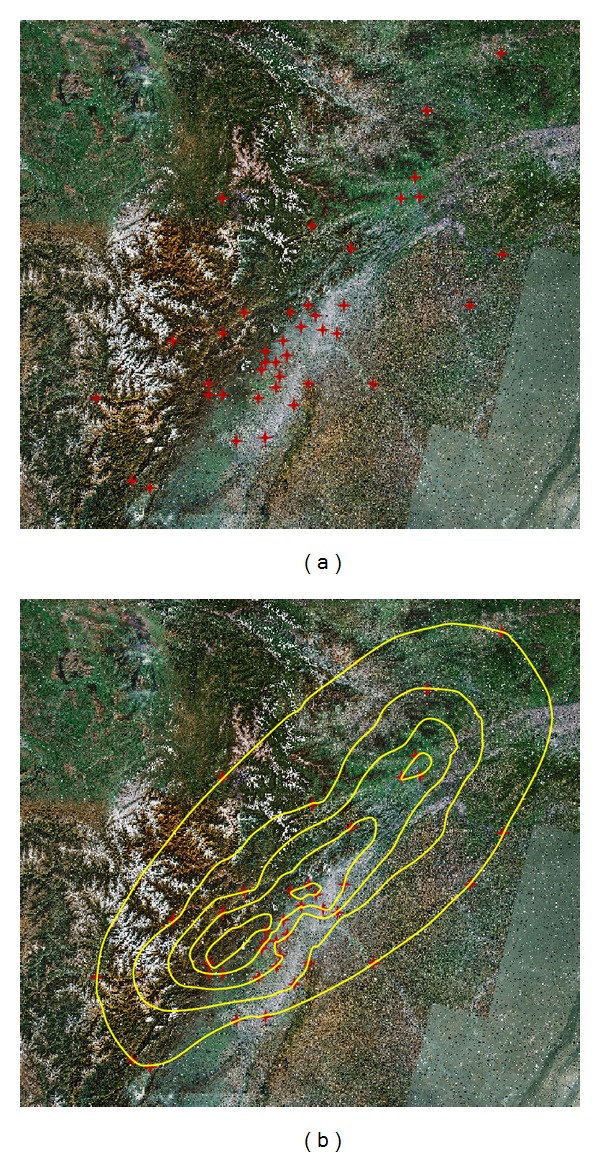
(a) Forty points labeled in remotely sensed satellite image; (b) isoseismal line based on forty discrete evaluation areas.

The number of the evaluation areas would affect the accuracy of the isoseismal line. As a comparison, this study added other twenty-five evaluation areas to draw the isoseismal line. They were Xiaojin County, Baoxing County, Pingwu County, and so forth.  Figure 5 shows the isoseismal line based on the forty evaluation areas.

#### 3.3.2. Seismic Intensity Map Rendering Based on GIS

**Figure 6:**
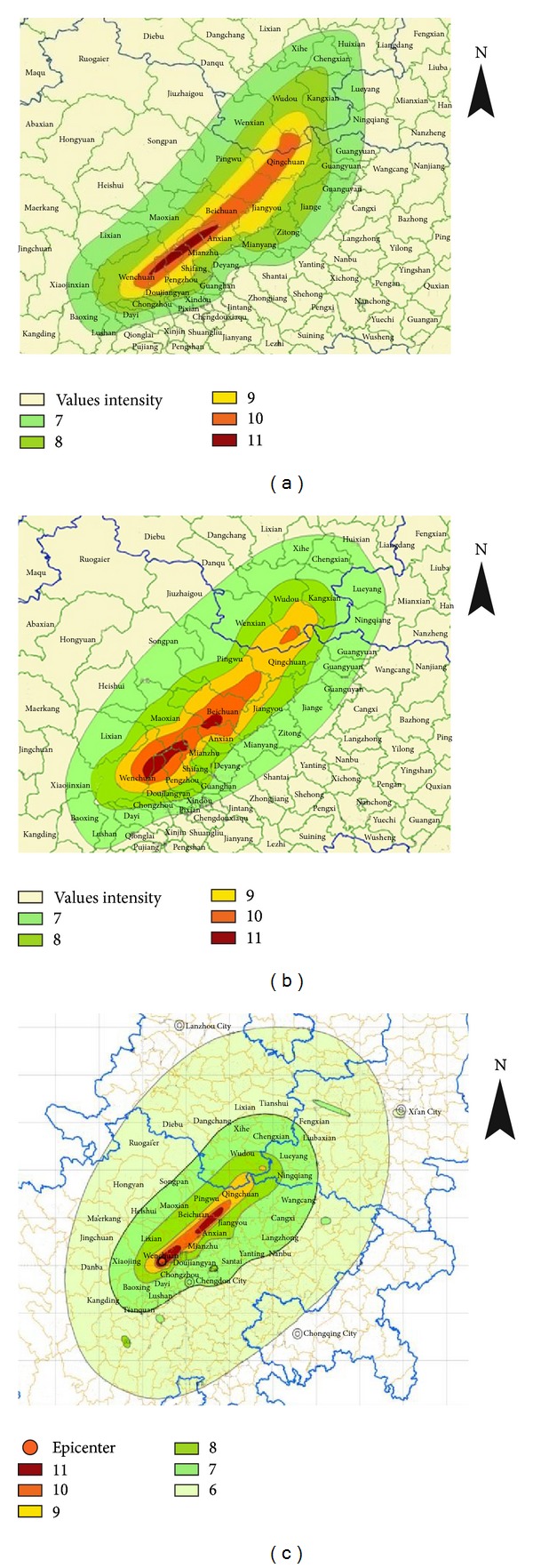
(a) Intensity map based on fifteen evaluation areas; (b) intensity map based on forty evaluation areas; (c) intensity map based on field investigation (published by Seismological Administration of China).

The visual output of seismic intensity value needs to convert the isoseismal line to render map. By overlaying the seismic intensity map and the administrative area map with GIS, it is clear to know the severity degree of the earthquake disaster in each region (Figure 6).

Overall, the three earthquake intensity maps in Figure 6 are very similar. The main damage areas are distributed along the Longmenshan fault zone. The intensity of earthquake center is eleven and distributed around Yingxiu and Beichuan County. However, there are still some differences among them. Firstly, Figure 6(b) with forty points is closer to the field investigation result in Figure 6(c) than Figure 6(a) with only fifteen points. The main reasons are that the seismic intensity map drawn from fewer evaluation areas is more dependent on the mathematical interpolation method, which would reduce the accuracy. Secondly, the recognition of seismic intensity in high intensity area is more accurate than that in the low intensity area. In high intensity area, the buildings are more vulnerable, and therefore the collapsed houses are easier to be recognized than the slightly damaged houses in low intensity area from remotely sensed images. Thirdly, the minimum intensity recognized in this study is seven. However, in the field investigation map, the minimum seismic intensity is six. The main reason is that the slightly damaged houses are hard to be recognized, especially in the remotely sensed images with low resolution.

## 4. Conclusions

This paper proposes an automatic recognition approach for seismic intensity based on the integration of spatial information technologies such as RS and GIS. It uses remotely sensed image to discover and retrieve the potential damage information caused by earthquake, which can automatically realize the fast extraction and recognition of the information on seismic intensity. In addition, GIS technology is applied to draw and render the seismic intensity map, which provides a visual way to query and output the information of seismic intensity.

The case study in Wenchuan Ms8.0 earthquake shows that the seismic intensity map recognized by the approach proposed in this paper is very similar to the real results investigated from the field area. However, the RS/GIS based method is much superior to the way of field investigation in time and resource expenditure. It can rapidly know the distribution of earthquake disaster, locate the strong seismic intensity area, and therefore win golden time for earthquake relief. Although the approach is only exemplified in Wenchuan Ms8.0 earthquake in China, it can be extended to a national or global level so as to recognize the seismic intensity in an effective, quick, and economical way. With the development of remote sense technology, more and more high-resolution images will be available in the near future; the RS/GIS-based approach proposed in this paper can play more significant roles in the automatic recognition of seismic intensity, especially in the cases that the transportation and communication are completely interrupted by earthquake.

## Figures and Tables

**Table 1:** Standard of seismic intensity division.

Seismic intensity	Condition of building collapse			Mean damage index	Earth's surface status
	Concrete	Multilayer brick	General houses		
VII	0.00	5.47%	6.00%	0.11–0.30	Soft land is cracked; most detached brick chimneys are moderately damaged; riverbank is collapsed.

VIII	0.00	12.91%	15.00%	0.31–0.50	Hard land is cracked; most detached brick chimneys are seriously damaged.

IX	5.40%	25.86%	33.00%	0.51–0.70	Hard land is cracked; most detached brick chimneys are collapsed; bed rock is cracked; landslide occurs.

X	24.73%	50.79%	88.00%	0.71–0.90	Landslide and rupture appear; most detached brick chimneys are collapsed from the root; bedrock arch is destroyed.

XI	50.00%	80.00%	95.00%	0.91–1.00	The earthquake ruptures continue long; a large number of landslides occur.

**Table 2:** Building damage rate in Wenchuan County.

Destruction category	Concrete	Multilayer brick	General houses	Earth's surface status
Collapse	21%	43%	90%	Landslides occur in large area; ground surfaces are fractured.
Destroy	40%	35%	9%	
Basic intact	39%	22%	1%	

**Table 3:** Results of seismic intensity recognition.

Region	Intensity
Wenchuan	X
Doujiangyan	X
Zundao	IX
Zagunao	VIII
Yinxiu	XI
Anxian	X
Jiangyou	X
Mianyang	VIII
Nanba	IX
Maoxian	VIII
Pengzhou	IX
Qingchuan	X
Hongbai	XI
Wangchang	VIII
Beichuan	XI
